# Process Modeling of Composite Materials for Wind-Turbine Rotor Blades: Experiments and Numerical Modeling

**DOI:** 10.3390/ma10101157

**Published:** 2017-10-05

**Authors:** Birgit Wieland, Sven Ropte

**Affiliations:** Institute of Composite Structures and Adaptive Systems, German Aerospace Center, Lilienthalplatz 7, D-38108 Braunschweig, Germany; Sven.Ropte@dlr.de

**Keywords:** rotor blade for wind turbine, composite materials, process modeling, temperature

## Abstract

The production of rotor blades for wind turbines is still a predominantly manual process. Process simulation is an adequate way of improving blade quality without a significant increase in production costs. This paper introduces a module for tolerance simulation for rotor-blade production processes. The investigation focuses on the simulation of temperature distribution for one-sided, self-heated tooling and thick laminates. Experimental data from rotor-blade production and down-scaled laboratory tests are presented. Based on influencing factors that are identified, a physical model is created and implemented as a simulation. This provides an opportunity to simulate temperature and cure-degree distribution for two-dimensional cross sections. The aim of this simulation is to support production processes. Hence, it is modelled as an in situ simulation with direct input of temperature data and real-time capability. A monolithic part of the rotor blade, the main girder, is used as an example for presenting the results.

## 1. Introduction

The production of rotor blades for wind turbines is still a predominantly manual process. In order to improve blade quality without a significant increase in production costs, tolerance management is combined with statistical process control. A process model with both analytical and numerical elements is developed, based on statistical production data and experiments. The investigation presented here focuses on the simulation of temperature distribution for one-sided, self-heated tooling and thick laminates. The model includes the properties of the heating system and the exothermic behavior of resin. The simulation is linked to experiments with varying process temperatures and laminate thicknesses.

**Figure 1:**
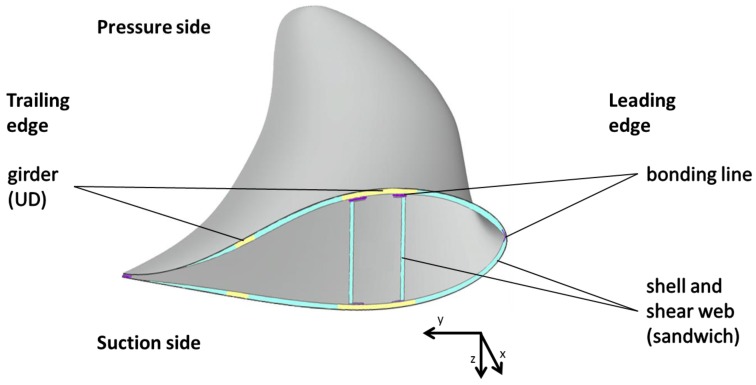
Typical cross section of a rotor blade according to [1,2,3].

Even if the geometry of rotor blades is not as complex as an airplane wing cover, different zones have to be taken into account. In Figure 1, a generic cross section of a rotor blade is shown. The main, girders (yellow), and possibly minor girders, are made of unidirectional glass or carbon-fiber reinforced plastics. Non-crimped fabric, pre-impregnated fibers or rovings can be used as raw materials according to the company-specific production process. As the girders take the main bending loads, the laminate quality of these parts is crucial. Torsional loads are carried by the shells and shear webs (turquoise). Therefore, a higher moment of inertia is required. In order to achieve this without unnecessary weight gain, sandwich material is used for shells and shear webs. Thin glass-fiber top layers cover a polyethylene terephthalate (PE/PET) foam core. Sometimes, in regions of higher loads, balsa core material is also used.

**Figure 2:**
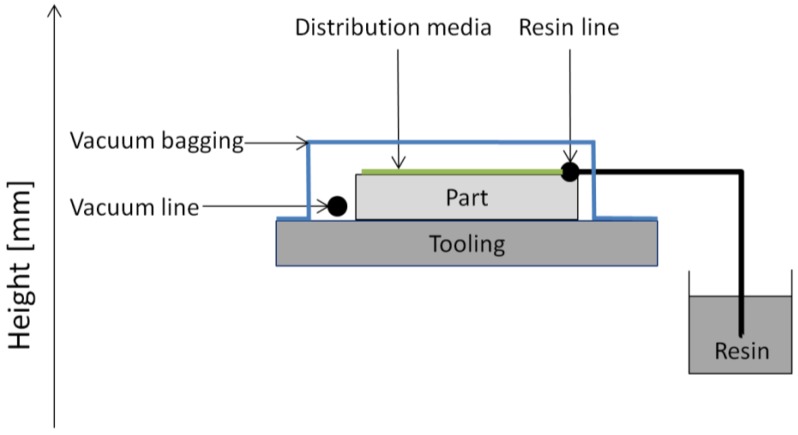
Vacuum-assisted resin infusion (VARI) procedure according to [4,5].

The girders are prefabricated parts and connected with the shells by the infusion process (Figure 2). Shells are joined to each other and with the shear webs in a bonding process with thixotropic adhesives for secondary bonding lines (purple).

A general structure of a rotor blade middle section, including the material distribution described, is summarized in Figure 1.

Figure 1 only shows the outer (tip) section of a rotor blade. In the root section are zones with wall thicknesses of up to 100 mm made of triaxial glass-fiber composite for the load transfer between blade and hub.

The production process of rotor blades includes the prefabrication of girders, shear webs, parts of root sections, and additional smaller parts. These are joined to the suction or pressure side shell, respectively, by a liquid resin infusion process. In most cases, the shear webs (one or more) are bonded to one shell and cured. Blind bonding is the final production step of the blade blank. Here, the shells and the shear webs are connected to a closed rotor blade.

The central production step for manufacturing fiber-reinforced plastics for wind-turbine applications is the infusion process. As each manufacturer has their own special features, Figure 2 shows a general overview of a vacuum-assisted resin infusion (VARI) process.

In Figure 2, a generic part of dry fabric is impregnated with epoxy resin. For rotor blades, in the majority of cases non-woven fabric is used. The infusion is driven by pressure differences between the low pressure in the cavity of the part (evacuated by vacuum line) and the resin reservoir under ambient pressure. The filling ratio, the fiber volume faction, is achieved by the equilibrium of forces between the resin reservoir and part compaction. For complete filling, the resin is spread by the resin line and a distribution media on the top surface of the component.

Due to the part’s large dimensions, tooling is one-sided, with an integrated heating system. The resulting challenge is achieving the temperature gradient between tooling and part surface, which becomes more significant with rising wall thicknesses and isolation materials.

The resins used are mostly epoxy-based. To achieve an acceptable occupancy time for tooling, they are cured at temperatures of about 80 °C. During this curing process, the epoxy resin’s reaction is exothermic. This poses a process risk that the resin’s limit temperature will be exceeded. Due to the laminate’s low thermal conductivity, this risk increases with the thickness of laminates, which need to be reduced. Through the prediction of process temperatures, thermal damage to parts due to the melting of auxiliary materials, process-induced deformations [6] or laminate characteristics [7], can be avoided.

Therefore, a simulation module for predicting the process temperatures in fiber-reinforced plastics with one-sided, heatable molds is required.

## 2. Theory

The curing process and its corresponding temperature profile is relevant for predicting residual stresses and process-induced distortions. For wind-energy laminates, one-dimensional modeling is not sufficient. In the thickness direction, the temperature distribution is not homogeneous as it is in thin laminates. The resulting heat profile depends on the characteristics of the heating system, including the thermal boundary conditions, fiber and matrix type. The main influencing parameters are the curing temperature and the thermal conductivity coefficients of the part and the tooling materials.

According to Zimmermann [8], exothermic energy released during the resin cure needs to be taken into account. In sum, the temperature profile of thick wind-energy laminates can be modeled by applying the heat equation. This method is also proposed by [9,10].

In the Fourier approach to the heat equation, the thermal conductivity coefficient is referred to as k and depends on the thermal conductivity, density and thermal capacity of the material used. T is the absolute temperature, and $\rho H_{R}\frac{d\alpha }{dt}$ represents the exothermic energy, with $H_{R}\;$ for the heat and $\frac{d\alpha }{dt}$ for the curing rate gradient.

The thermal model for a three-dimensional part of fiber-reinforced plastic is:

(1) $$\frac{\partial }{\partial x}\left(k_{x}\frac{\partial T}{\partial x}\right)+\frac{\partial }{\partial y}\left(k_{y}\frac{\partial T}{\partial y}\right)+\frac{\partial }{\partial z}\left(k_{z}\frac{\partial T}{\partial z}\right)+\rho H_{R}\frac{d\alpha }{dt}=\frac{\partial T}{\partial t}$$

The conductivity vector can be calculated, according to Twardowski et al. [11], by using the Springe–Tsai model. For the remaining parameters, the mixing rule can be used. To solve the heat equation, the heating system is modelled and, on the upper side of the part, convection boundary conditions are applied.

As mentioned, the exothermic energy release has a major effect [12]. It is triggered by a chemical reaction and is linked to the current change of curing degree at the moment of observation. Therefore, the simulation includes a cure module. This curing process of the thermoset material utilized is modelled according to the phenomenological model of Kamal and Sourour [13,14,15,16,17].

To characterize the interdependence between expected processing temperature and reaction velocity, Guo et al. [18] use a numerical model. The majority of references (e.g., [19,20]) are focused on autoclave curing processes for thin laminates with one-dimensional abstraction. In autoclave processes, the boundary conditions are significantly more stable compared to wind-energy applications, but both inherit the exothermic effect as a critical element. To minimize its impact, Oh et al. [20] use a three-dimensional formula in order to find an optimized tempering cycle. Mesogitis [21,22] enhanced this approach with a stochastic simulation of an initial curing ratio, activating energy and reaction sequences. For one-side heatable tooling, the scatter in mold temperature, conductivity coefficients and surrounding temperatures influences the reaction velocities as well. Additionally, Wang et al. [23] identify curing temperature and heating rate as impact factors on curing uniformity.

To conclude, a model of process temperature for thick wind-energy laminates must include the exothermic reaction as well as material and tooling parameters.

## 3. Experiments: Influence Parameters

The physical model of the temperature distribution in thick laminates includes the input parameter, identified through a literature review. For rotor-blade components out of series production (Nordex Energy GmbH, Hamburg, Germany), the following experiments and measuring campaigns ascertain additional disruptive factors and input parameters.

### 3.1. Rotor-Blade Production

**Figure 3:**
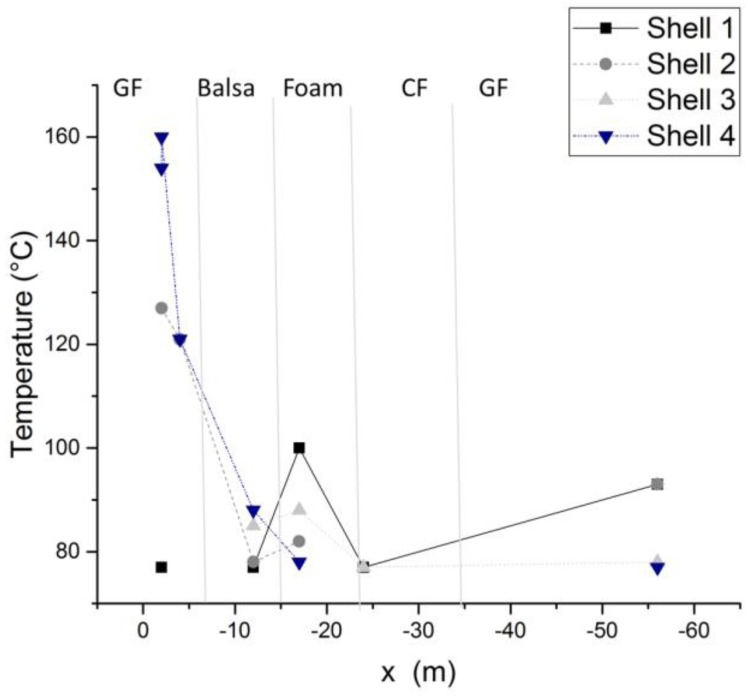
Maximum tooling surface process temperatures

An adequate model for process temperature takes account of all factors influencing rotor-blade production. Therefore, the maximum temperatures on the tooling surface and the temperature profile on the part surface were measured. Figure 3 shows the maximum temperature occurring during the curing process of rotor-blade shells. The values are measured by thermos labels on the tooling side to avoid process risks. With the measurements in Figure 3 the impact of the laminate structure on the process temperature can be identified. The highest temperatures are reached below the thick glass-fiber (GF) laminate in the root section. The area of sandwich material (Balsa and Foam) does not indicate such an exothermic reaction. The precured main girder made of carbon fiber (CF) has no influence on the heating system. The thin shell layer in the tip of the rotor blade shows some exothermic overshots.

**Figure 4:**
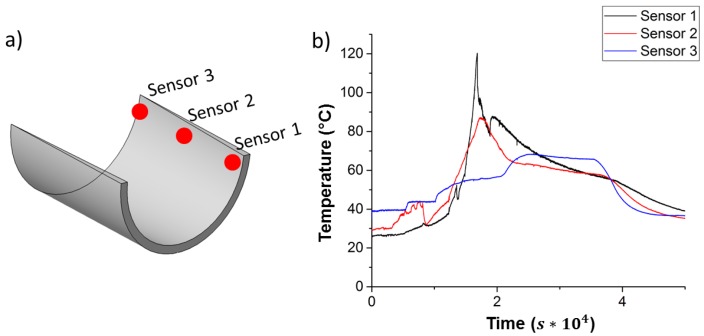
Temperature profiles for root section; (**a**) sensor positions; (**b**) measurement data.

Figure 4 shows the measurement data of thermocouples on top of a root section with vacuum bagging. The lay-up of the root section contains varying numbers of non-crimped glass-fiber layers. For the connection area to the hub, a very thick laminate (up to 100 mm) is needed. Sensor 1 is located at this radius (x = -1 m). The number of layers within the root section decreases to one layer at a radius x = -4 m. Sensor 3 is placed here. Sensor 2 is equidistant between sensors 1 and 3, laterally as well as for a number of layers. Figure 4b shows temperature distribution on the part surface, including the thermal energy caused by the exothermic reaction. It demonstrates the influence of higher part thicknesses on the exothermal peak temperature.

### 3.2. Laboratory

**Figure 5:**
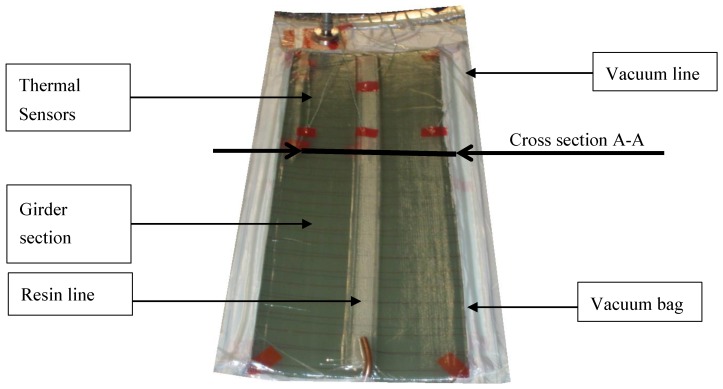
Infusion set-up for girder sections.

Experiments in the laboratory give an opportunity to reduce the number of external disturbance factors and to undertake detailed analysis inside a part. To generate verification data, sections of a main girder are built in an experimental, wind-energy typical, mold. This tooling is a female geometry of a rotor-blade section with scale 1:2 and it is equipped with a fluid-heating system. The water pipes are integrated in loops in the tooling wall. The heating temperature, measured at the return outlet, is set by an extra controller. The test set-up for the part is depicted in Figure 5.

**Figure 6:**
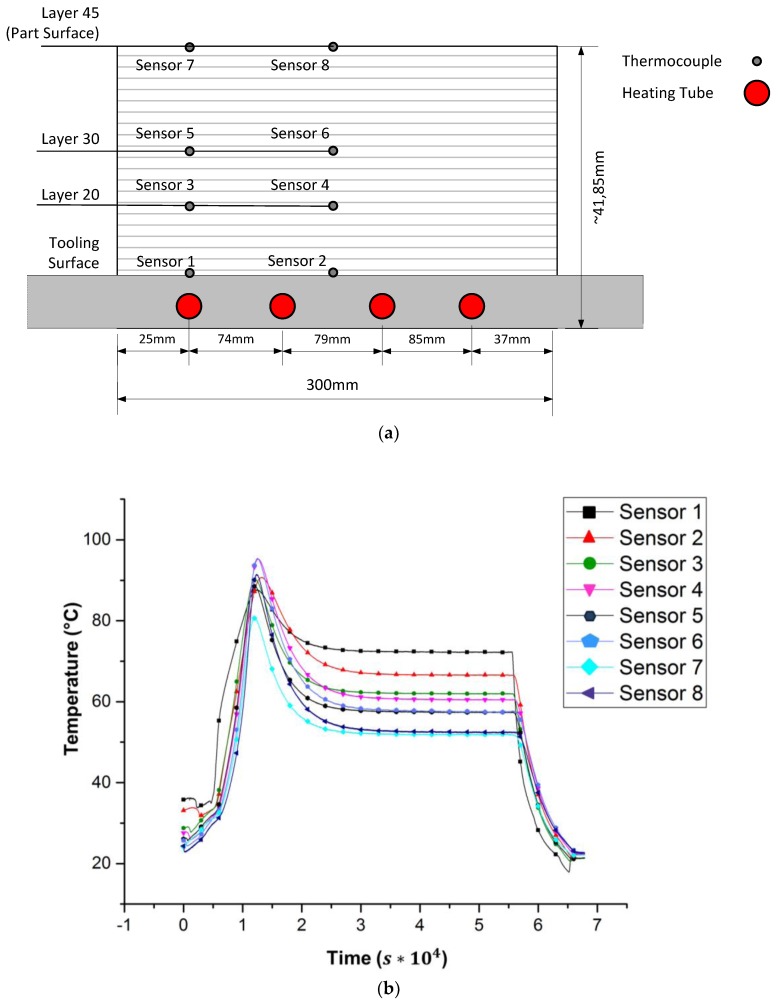
Temperature profiles during the curing process; (**a**) senor position; (**b**) measurement data.

Figure 5 shows the infusion of a 45-layer thick unidirectional part with a central resin line. The fibers were orientated 0° in the component lengthwise direction; the areal weight of the non-crimped fabric used was 1200 g/m³. For infusion, the resin “Airstone 880” was used. The infusion started at room temperature. For curing, a heating temperature of 75 °C was chosen. In the experiment, a dense network of thermocouples was employed, and these were located within the laminate (Figure 6a). The measuring points were separated within the thickness direction of the part, as well as in a lateral orientation. Figure 6 shows the results for sensors 1 and 2, located on the tooling surface (layer 0), sensors 3 and 4 on layer 20, sensors 5 and 6 on layer 20, and sensors 7 and 8 on the part surface with layer 45.

In Figure 6b, the temperature profiles of six thermocouples over time are shown. The measurement starts shortly before the beginning of the infusion process (time $t=0.3\times 10^{4}\;s$). After an infusion time of $t=0.7\;\times 10^{4}\;s$, the temperature rises because of the heating temperature and exothermic reaction. The maximum peak temperature is reached at $t=1.3\;\times 10^{4}\;s$. The part remains at 50 °C up to 72 °C until cooling down at time $t=5.6\;\times 10^{4}\;s$, and ends up reaching ambient temperature.

The temperature profiles demonstrate the temperature overshoots as a result of the exothermic reaction. Analyzing the plots’ temperature, gradients in thickness and lateral direction are visible. The exothermic effect is at its maximum in the middle of the part (sensors 3, 4, 5, and 6). On the component surface (sensors 7, 8), convectional heat transfer losses reduce the average curing temperature. The difference between temperatures in the middle position compared to the results at the part’s left side in Figure 6 is based on the location of thermal sensors relative to the heating loops in the tooling wall. Accordingly, sensors 1, 3, 5, and 7 are positioned directly upon such a tube, while the other measuring points are located half way between two loops. The average curing temperature for sensors positioned above the heating tubes is higher, e.g., for comparing sensors 1 and 2. The same effect can be observed for the temperature gradient in the thickness direction. The heating tube effect leading to higher temperatures decreases with increasing layer number.

Results of the experiments provide the boundary conditions for the following model of process temperature distribution in thick laminates.

## 4. Model

As a result of the theoretical background and influence parameters identified, within the next section a numerical model for predicting real-time calculation and process temperature, respectively, will be introduced.

### 4.1. Physical Model

**Figure 7:**
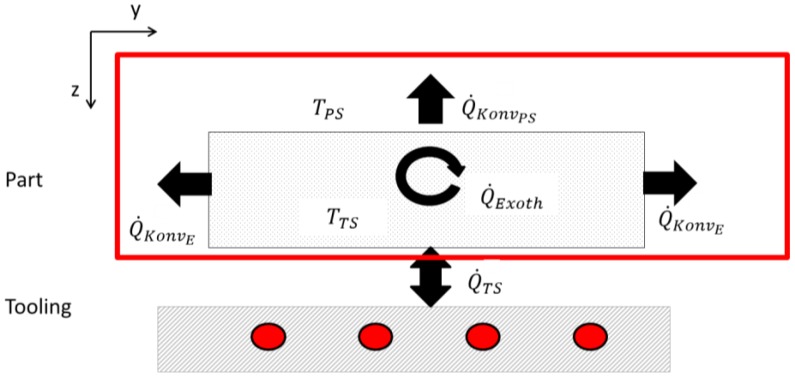
Schematic sketch of thermal fluxes.

According to the chosen approach, the two-dimensional heat equitation, Figure 7 shows the thermal fluxes during the curing of composite materials on one-side heatable toolings. The thermal fluxes are here calculated with

(2) $${\dot{Q}}_{z}=\frac{\partial }{\partial z}\left(k_{z}\frac{\partial T}{\partial z}\right)$$

The first heat transfer between the mold and part, ${\dot{Q}}_{TS}$, with the corresponding temperature,$\;T_{TS}$, transfers the thermal energy of heating to the component. For fluid-heating systems, it acts in both directions. For temperature rises and the remaining phase during the curing cycle, the heat transfer occurs in a negative z-direction. During the exothermic reaction and its corresponding temperature increase, as well as during the cooling-down phase, the thermal flux is oriented in a positive z-direction. This process is closely linked to the laminate-up and its thermal characteristics, as was shown in Figure 3.$\;\;T_{TS}$ is determined by control of the heating system, the isolation effect of the lay-up, and the exothermic reaction. The component-internal heat source, ${\dot{Q}}_{Exoth}$, has an additional effect on the thermal system. In formula (1), ${\dot{Q}}_{Exoth}$ is described with

(3) $${\dot{Q}}_{Exoth}=\rho H_{R}\frac{d\alpha }{dt}$$

This results from the exothermic curing reaction of epoxy resins, and is determined by characteristic values of the resin, the fiber volume fraction and cure rate.

Interaction with the environment is abstracted by the convection heat fluxes on the part surface, ${\dot{Q}}_{Konv_{PS}}$, and on part edges, ${\dot{Q}}_{Konv_{E}}$. Also, the related temperatures, $T_{PS}$, are measurable. Here, ambient temperature, surface area and possible isolation measure have an impact. Together with the principle of the conservation of energy, Equation 1, material parameters and boundary conditions, the system described can be translated into a mathematic expression. For simulating the temperature distribution, a numerical model, based on this physical system, needs to be generated. Therefore, some assumptions are required. The length of a main girder is significantly higher than its width. Hence, it can be assumed that there are no interactions between the cross sections. This allows good approximation by a two-dimensional numerical model. The second assumption is that the surface of the part edges is small compared to the top surface. Therefore, ${\dot{Q}}_{Konv_{E}}$ can be neglected. In the first stage of process temperature simulation presented here, the control algorithm as well as the convection heat transfer is not modelled. As the experiment has already shown, this paper focuses on unidirectional laminates. Thus, the measured temperature distributions for $T_{PS}$ and $T_{TS}$ will be used as boundary conditions. The discontinuity of the heating system is approximated by user-defined oscillation of $T_{TS}.$

### 4.2. Numerical Model

**Figure 8:**
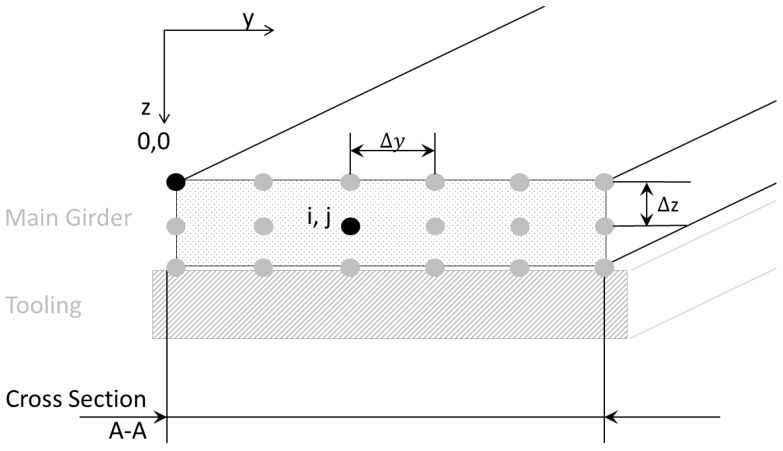
Discretized cross section.

For numerical modelling, the cross section is discretized by equal rectangle elements. At the nodes $a_{0,0},\ldots a_{i,j},\ldots a_{n,m}$, the curing degree and temperature for each time step are calculated. The resolution is user-defined by Δy and Δz. The schematic set-up is shown in Figure 8.

As is appropriate for discretization parameters, the number of nodes is determined automatically. This number defines the scope of coefficient matrices for a one-step procedure for solving the 2D-Fourier-approach for the heat equation. Together with specific resin parameters, boundary conditions, initial temperature and curing distributions, an implicit Euler return mapping scheme can be established. Until the user-defined time steps are reached, the solving cycle between temperatures and curing state is iterated. Analytically relevant data are stored for each iteration step in documentation files.

**Figure 9:**
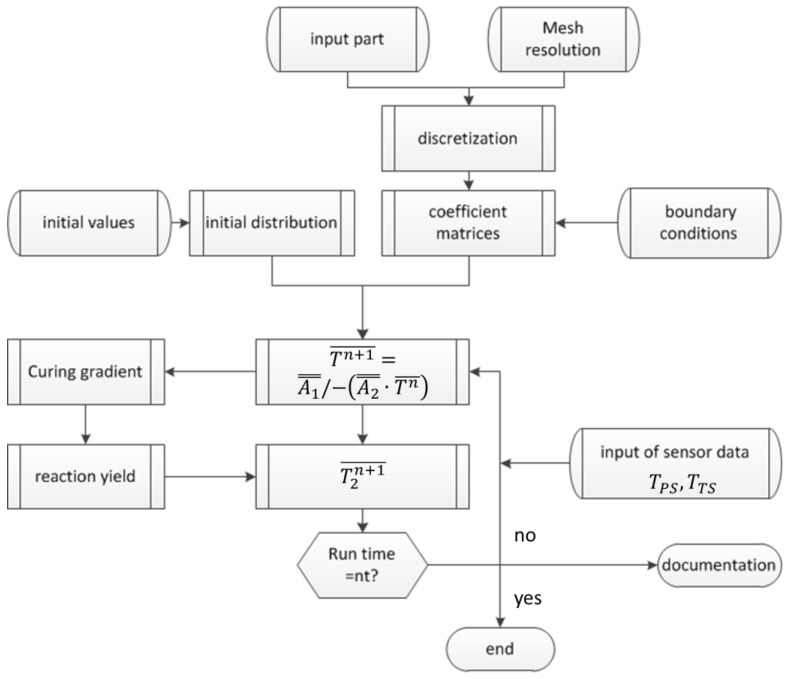
Unified model language (UML) diagram of the process temperature simulation algorithm.

In Figure 9, the unified model language (UML) diagram for the simulation summarizes the procedures for predicting temperature and curing degree.

### 4.3. Results of Numerical Simulation

**Figure 10:**
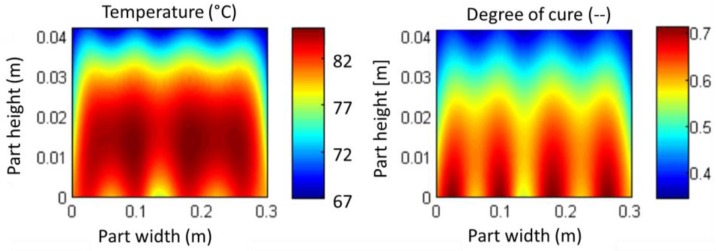
Temperature and curing ration for section A–A after *t* = 10,640 s.

The numerical model developed includes two main modules: the curing model and the solution of the heat equation. With the input of surface temperatures $T_{PS}$ and $T_{TS}$, the Fourier formula can be solved. The given temperature distribution enables the curing module to calculate the reaction rate and the released enthalpy. This leads to an explicitly calculated increase of the temperatures for each node per time step. The model allows the simulation of temperature and degree of cure distribution over time in high resolution, depending on given material parameters and surface-temperature history. The temperature of a single node over time can be displayed as well as the temperature and curing ratio distribution over the whole cross section for special points in time, as in Figure 10.

On the left side of Figure 10, the temperature distribution is shown shortly before maximum peak temperature is reached. Gradients have not equalized due to inhomogeneous heating. The hotspots are in the middle of the part, corresponding to measurement results in Figure 6. The difference between the hotspots and component surface reaches up to 15 K. This is critical, as it leads to a gradient in the curing degree, shown on the right side of Figure 10. The chemical reaction is influenced by time and temperature. So, for the chosen temperature cycle, the curing of resin starts in the regions above the heating tubes. This engenders a risk of developing internal stresses and process-induced deformations. The temperature and curing distributions for special points in time give an opportunity to identify local hotspots and to examine geometric deformations in thick laminates.

**Figure 11:**
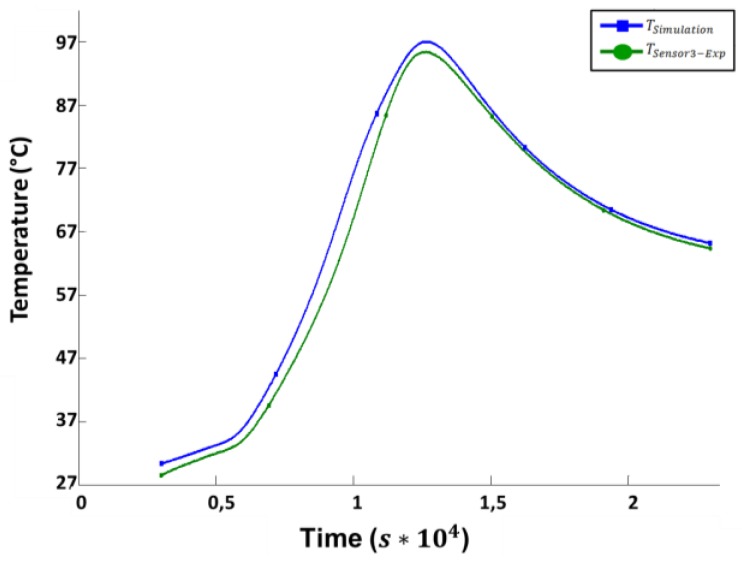
Comparison between simulated and measured data for sensor 3.

The total effect of the exothermic temperature peak can be seen in Figure 11. The diagram shows the temperature profile for sensor 3 and the corresponding simulation results.

## 5. Discussion

**Figure 12:**
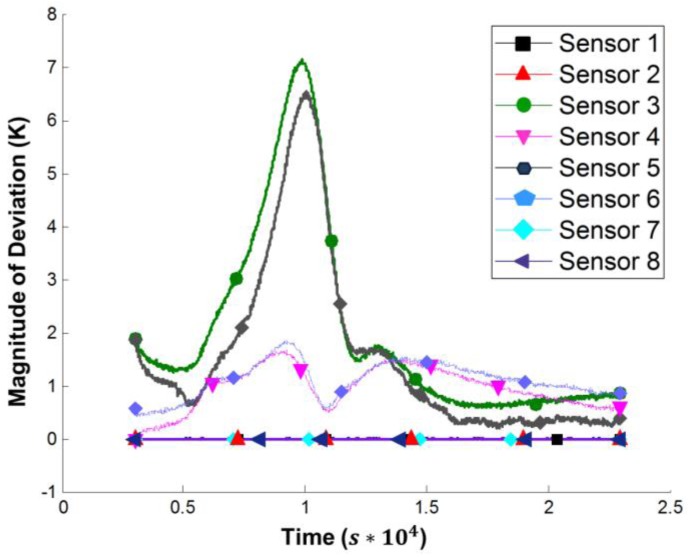
Magnitude of deviation between simulation results and measurement data.

The simulation is verified by comparison with the experimental data. For temperature distributions in cross sections, a high-resolution measurement is impossible. Hence, the verification is located at the measurement points shown in Figure 6a. As shown in Figure 11, the temperature evolution over time is acquired experimentally and calculated by the simulation for each sensor position. For evaluation, the differences that occur between measured and calculated temperatures are plotted in Figure 12.

Sensors 1, 2, 7 and 8 provide input data for the simulation. Therefore, they show no deviation. The discrepancies for the simulated, virtual sensors 3 and 4, positioned in the middle of the part, are smaller than 2 K. The data of sensors 5 and 6 show a significantly higher deviation. Due to the infusion process, the initial degree of curing varies over the cross section, especially in the thickness direction. So one reason for this high deviation is a temporal out-phasing by a relatively large temperature gradient over time. This deviation can also be seen in Figure 11 for sensor 3. A potential solution may be a model for the distribution of the initial degree of curing.

## 6. Conclusions

This paper demonstrates the utility of a physical model to predict temperature distributions in thick laminates for wind-turbine rotor blades. The results enable an improvement in the curing process in order to minimize process-induced deformations and process risks through temperature overshots. Temperature management can be adapted to achieve a more homogenous curing process without unwanted temperature peaks. Indirectly, this will lead to a reduction of process-induced deformations. Additionally, the simulation gives an opportunity to optimize curing cycles in order to reduce production time, costs, and the occupancy time of the rotor-blade main mold.

Further research is planned on the modelling of supplementary laminate lay-ups, such as sandwich areas, as well as a link to a numerical simulation to control fluid heating. Elements of present research include deeper analyses of residual stress distribution for thick laminates, as well as temperature-induced tooling deformations. Combined with process simulation for fiber volume fraction and other modules, a complete tolerance simulation system for the production of rotor blades will be implemented.
